# Mass anti-malarial administration in western Cambodia: a qualitative study of factors affecting coverage

**DOI:** 10.1186/s12936-017-1854-4

**Published:** 2017-05-19

**Authors:** Christopher Pell, Rupam Tripura, Chea Nguon, Phaikyeong Cheah, Chan Davoeung, Chhouen Heng, Lim Dara, Ma Sareth, Arjen Dondorp, Lorenz von Seidlein, Thomas J. Peto

**Affiliations:** 1 Centre for Social Science and Global Health, University of Amsterdam, Amsterdam, The Netherlands; 2 Amsterdam Institute for Global Health and Development, Amsterdam, The Netherlands; 3 Mahidol Oxford Tropical Medicine Research Unit, Mahidol University, Bangkok, Thailand; 4 Academic Medical Centre, University of Amsterdam, Amsterdam, The Netherlands; 5 National Center for Parasitology, Entomology and Malaria Control, Phnom Penh, Cambodia; 6 Centre for Tropical Medicine and Global Health, Nuffield Department of Clinical Medicine, University of Oxford, Oxford, UK; 7 Battambang Provincial Health Department, Krong Battambang, Cambodia

**Keywords:** Malaria, Qualitative, Attitudes, Social factors, Community engagement, Mass drug administration, Coverage, Cambodia

## Abstract

**Background:**

Mass anti-malarial administration has been proposed as a key component of the *Plasmodium falciparum* malaria elimination strategy in the Greater Mekong sub-Region. Its effectiveness depends on high levels of coverage in the target population. This article explores the factors that influenced mass anti-malarial administration coverage within a clinical trial in Battambang Province, western Cambodia.

**Methods:**

Qualitative data were collected through semi-structured interviews and focus group discussions with villagers, in-depth interviews with study staff, trial drop-outs and refusers, and observations in the communities. Interviews were audio-recorded, transcribed and translated from Khmer to English for qualitative content analysis using QSR NVivo.

**Results:**

Malaria was an important health concern and villagers reported a demand for malaria treatment. This was in spite of a fall in incidence over the previous decade and a lack of familiarity with asymptomatic malaria. Participants generally understood the overall study aim and were familiar with study activities. Comprehension of the study rationale was however limited. After the first mass anti-malarial administration, seasonal health complaints that participants attributed to the anti-malarial as “side effects” contributed to a decrease of coverage in round two. Staff therefore adapted the community engagement approach, bringing to prominence local leaders in village meetings. This contributed to a subsequent increase in coverage.

**Conclusion:**

Future mass anti-malarial administration must consider seasonal disease patterns and the importance of local leaders taking prominent roles in community engagement. Further research is needed to investigate coverage in scenarios that more closely resemble implementation i.e. without participation incentives, blood sampling and free healthcare.

## Background

The spread of multidrug-resistant *Plasmodium falciparum* is a serious threat to current global malaria prevention and control efforts [1]. In parts of Southeast Asia, artemisinin resistance has emerged and urgent action is needed to prevent resistant parasites from spreading across Asia to Africa [1–3]. Such a scenario—which would be consistent with the past expansions of other resistant strains—has catastrophic implications for the region that suffers the greatest burden of malaria-related morbidity and mortality [4, 5]. For this reason, the World Health Organization described the elimination of *P. falciparum* as an urgent priority in the Greater Mekong Subregion (GMS) [6].

Efforts to eliminate *P. falciparum* in the GMS have therefore been intensified. One initiative, targeted malaria elimination (TME), combines conventional malaria prevention and control activities [such as strengthening the network of village malaria workers (VMWs) to provide appropriate case management and distribute long-lasting insecticide treated bed nets (LLINs)], with the mass administration of artemisinin combination therapy (ACT) in areas with an asymptomatic reservoir of malaria. The mass drug administration (MDA) component of TME entails delivering a curative anti-malarial dose to all individuals within a community, irrespective of malaria infection (and without reliance on diagnostic tests) to interrupt local transmission. Parallel research into TME is underway in Myanmar, Vietnam, Cambodia, and Laos to determine the potential of TME as a tool for *P. falciparum* elimination in areas of suspected or proven artemisinin resistance.

**Fig. 1:**
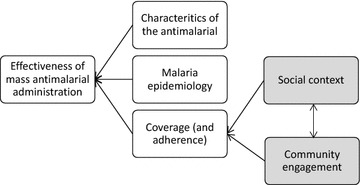
Factors affecting the effectiveness of mass anti-malarial administration

The complete interruption of local *P. falciparum* transmission through mass administration of an ACT can only be achieved with high level of coverage in the targeted population [7, 8]. Although the required level of coverage depends on the local epidemiology and intensity of transmission, this, along with the characteristics of the ACT, is crucial for the success of the MDA [9]. The local social and cultural context, which operates through, for example, people’s understandings of the disease in question and their risk of being affected, perceptions of the benefits of taking medication when healthy, and local political dynamics, affects MDA coverage [10, 11] (Fig. 1). To promote coverage, MDA programmes have undertaken a range of activities, such as employing local people, including community health workers and other field workers, seeking support from village leaders and offering health education. Broadly, these activities are often termed community engagement [12].

To date, only one qualitative study in Myanmar [13] has explored specifically how the local social context and community engagement activities influence coverage of (and adherence to) mass anti-malarial administration in Southeast Asia. Understanding this across other social contexts is necessary to inform future implementation of MDA for *P. falciparum* elimination in the GMS. This article explores the local community’s attitudes and behaviours towards the MDA, addressing questions such as: how do local understandings and experiences of malaria and prevention/control interventions influence people’s response to MDA? What role did the community engagement activities play in the uptake of MDA? How are other aspects of the social and cultural context relevant?

## Methods

### Setting

#### The clinical trial

Data were collected alongside a randomized controlled trial of TME, which included MDA, conducted during 2015–16 in Samlout District, Battambang Province, Cambodia. Five villages were selected based on evidence of local *P. falciparum* transmission from screening surveys and local treatment records: three villages received MDA in summer 2015. The study evaluated the effectiveness of treating entire villages with three, monthly rounds of dihydroartemisinin piperaquine, (DHA–PPQ), at the time, the first-line anti-malarial. This artemisinin-based combination is well-tolerated, but participants were advised of potential side-effects during meetings and on the informed consent form. Study staff administered the ACT over three days as directly observed therapy. To compensate participants for the opportunity costs of taking a day out from their (agricultural) work, they were given a cash payment of KHR 10000 (~US$2.5) for participation. Study participants also received free basic health care from a study physician who resided in the villages during the trial. Prior to the first round of MDA, and then at three-monthly intervals for 1 year, cross-sectional surveys were conducted to collect blood to determine the asymptomatic prevalence of malaria by ultra-sensitive polymerase chain reaction (uPCR). The uPCR method necessitated a 3 ml venous blood sample from adults and 0.5 ml sample from children aged under 5 years of age. Local malaria treatment records were collected throughout the study period [14–16].

#### Local social context

Samlout District is located along the Thai border and above the Cardamom Mountains. Most families are involved in agriculture and then main crops are cassava, corn, beans, vegetables, and rice. Younger adult men often travel to local forests to collect vines, fruit, wood, and to hunt. Many villagers migrate to other provinces or to Thailand for seasonal work.

Samlout has been a site of heavy fighting over the past 50 years, since an uprising against forced rice purchases by the government of King Norodom Sianhouk was put down by the army in 1967. Following the coup of 1970, many locals joined the Khmer Rouge and other groups fighting against the Khmer Republic during the civil war of 1970–75. During the period of Democratic Kampuchea (DK), 1975–79, large numbers were forced from the cities to the Battambang countryside and put to work in rice production many of whom died from diseases and starvation. Following the fall of the DK in 1979, the Khmer Rouge fled to western Cambodia and fighting continued throughout the 1980s and early 1990s. Many locals escaped to refugee camps in Thailand and others were caught up in the fighting. During these years, armed factions took to the forests. Following the civil war the population grew and new villages were established.

Samlout District also forms part of the Thai–Cambodia border region that was more recently targeted as a priority area for the containment of artemisinin-resistance [17]. This programme of activities included large-scale recruitment and training of village malaria workers (VMWs); the distribution of effective anti-malarials, rapid diagnostic tests, long-lasting insecticide-treated bed nets (LLINs) and hammock nets to stable and mobile/migrant populations; and engagement with private drug sellers to combat counterfeit and substandard anti-malarials, and to enforce the ban on the sale of anti-malarial monotherapies [17].

#### Epidemiological context

Battambang is an area of unstable malaria transmission and has seen a decline in cases of clinical *P. falciparum* malaria over the past 15 years [18]. Although malaria incidence has fallen since 2003, *P. falciparum* parasites in the area have become resistant to artemisinins and partner drugs used in ACT [2, 5, 19, 20]. Village Malaria Workers are present in most villages and are trained and supervised by the health department to diagnose and treat clinical malaria. Clinical and asymptomatic malaria infections are associated with travel to forests and a large proportion of local transmission may occur outside the villages [21, 22].

### Data collection

With the aim of increasing the reliability of findings, a range of qualitative data collection activities were undertaken with various respondent types. This enabled triangulation and lessened the potential bias of one particular method or respondent type. Conducting extended fieldwork in the communities, which included some informal conversation with villagers, also enabled the field staff to observe villagers’ behaviours towards the intervention.

The methods included semi-structured and in-depth (individual) interviews, focus group discussions (FGDs), exit-poll (a one-page questionnaire administered to participants on leaving community engagement activities). The qualitative data were collected by two trained field workers, fluent in Khmer and English, who were resident in the field site for 1 year.

Semi-structured interviews (SSIs) were conducted with around 10 respondents from each village (30 in total) selected at random from a list of de facto adult residents recorded by a household census conducted immediately prior to the study. The census recorded all household members irrespective of their stay in the village and was used for the purpose of defining coverage for the clinical study. A random sample was chosen because TME is a community-wide approach and this enabled the research to team to collect an overall impression of the community response to the intervention. These respondents were interviewed at around seven days after rounds one, two and three of the mass anti-malarial administration. The first interview was audio-recorded, transcribed and translated. Detailed notes were taken during interviews two and three. In-depth interviews (IDIs) were also conducted with TME staff members involved in implementing the project (and community engagement activities) (n = 5, all team members available at the time). Community members who refused to participate or who did not complete all three doses in all three rounds were also interviewed: respondents were selected randomly from study records and five were interviewed. All the individual interviews took place at the respondent’s home or in the immediate vicinity.

Focus group discussions with women and men from the TME villages, particularly those work in the local forests, an established risk factor for malaria infection. To make contact with respondents from these typically hard-reach groups who work in local forests, FGD participants were purposefully selected by the village leaders, who acted as gatekeepers. Focus group discussions were held in the compounds of the village leaders.

To gain a rapid and basic appreciation of villagers’ comprehension of community engagement activities during the study, exit questionnaires were administered to 30 respondents. This occurred at major community meetings when the study was explained and every tenth person leaving the event was selected as a respondent. On leaving the events, respondents were asked six questions covering basic demographic details (age group, village of residence) and about the basic details of the TME study: the purpose of the study, how long the study would last and what activities would be undertaken.

Interview topics included experience and understandings of malaria, anti-malarials and the TME project (including the community engagement activities). Focus groups also explored issues of the movement of forest-goers. Interviews with TME staff also covered the challenges associated with undertaking the project and the community engagement activities. The exit questionnaires included questions about basic details of the project, such as its aim and activities.

During the project, the field staff also recorded their observations of study activities and community engagement. This included, specifically, taking notes based on meetings with village leaders (n = 10) that were conducted during the design phase of the community engagement approach.

### Data processing and analysis

The SSIs (after round one), IDIs and FGDs, when consent was given, were audio recorded and transcribed verbatim and translated in English. For quality control a 10% sample of the SSIs were re-transcribed and re-translated by an independent translator then compared with the original transcriptions and translations. After this process, no significant inconsistences were found that warranted further revision of the transcriptions and translation. Field staff were also regularly debriefed by CP, TP, RT.

Transcripts and field notes underwent qualitative content analysis using a qualitative data analysis software package (QSR NVivo 10). Data analysis combined inductive and deductive elements: analytical categories were developed from the initial research questions and also emerged during the analysis process. The first author, therefore, conducted line-by-line coding of all data using a code book that was in-part pre-developed in light of the original research questions but flexible enough to allow the addition of inductive codes as required. Analysis continued by exploring the patterns of the codes across the transcripts/data sources, identifying exceptional cases and examining differences in the coded text. The results of preliminary analysis was discussed with the field staff during and immediately after the period of data collection. The content of the codes were used to develop the themes that are presented in the results.

## Results

With regard to attitudes to the MDA, key themes identified in the interviews and field notes included villagers’ understanding of malaria and malaria prevention, the community engagement that was delivered alongside the clinical study, the timing of the MDA and health complaints that were attributed to the anti-malarial. These factors are elaborated below. Table 1 contains information on the SSI respondents, particularly their participation across the three rounds of MDA: of the 30 who participated in round one, 15 participated in round two and 19 in round three (though several respondents could not be re-interviewed because of flooding).

**Table 1:** SSI respondent characteristics and reported reasons for non-participation in rounds 2 and 3 of mass anti-malarial administration

Village	Sex	Age group	Round 2	Round 3
Peam Ta	F	50–60		Unknown
Peam Ta	M	30–40	Forest visit	Unknown
Peam Ta	M	20–30		
Peam Ta	F	20–30	“Side effects” after round 1	
Peam Ta	F	40–50	“Side effects” after round 1	“Side effects” after round 1
Peam Ta	M	40–50	“Side effects” after round 1	“Side effects” after round 1
Peam Ta	F	60–70	“Side effects” after round 1	“Side effects” after round 1
Peam Ta	F	30–40		
O Treng	F	50–60	“Side effects” after round 1	Excluded on health grounds
O Treng	F	40–50	Excluded on health grounds	Excluded on health grounds
O Treng	M	30–40		Partial - forest visit
O Treng	F	30–40		
O Treng	F	30–40		
O Treng	F	30–40		
O Treng	F	18–20		
O Treng	F	30–40	Ill	
O Treng	F	30–40	Absent	
O Treng	F	20–30		
O Treng	F	50–60	“Side effects” after round 1	Inaccessible due to flooding
O Treng	F,M	40–50; 50–60	Inaccessible due to flooding	Inaccessible due to flooding
Veal Rolueum	M	30–40		
Veal Rolueum	F	50–60		
Veal Rolueum	M	30–40	Forest visit	
Veal Rolueum	M	50–60		
Veal Rolueum	F	50–60	“Side effects” after 2 doses	
Veal Rolueum	F	50–60	Unknown	Excluded on health grounds
Veal Rolueum	M	30–40		
Veal Rolueum	M	60–70		
Veal Rolueum	F	50–60	“Side effects” from round 1	
Veal Rolueum	M	50–60		

### Malaria prevention

Respondents were generally familiar with malaria as a symptomatic illness. Symptoms were particularly recognized as fever, shivering and headache, with more scattered references to sweating, nausea/vomiting and tiredness or lethargy.

*I:…What are the symptoms of malaria?*

*FGD4: Yes, for malaria, its first symptom is headache, yes headache when getting fever (Interruption from FGD1: headache [and] a sensation of chill in body) then starting with chills.*

*FGD1: Hotness (temperature increasing in body) [and] sweating.*

*FGD4: It’s hot [and] when we talk about sweating, it’s not like good sweating as usual, [but] it’s a sticky sweating. Yes, it’s wet [and] sticky. Yes, it’s hot, severe headache and start with chills. Yes, that’s chills. Yes, what we remember [from the past/experience] these are [symptoms] of malaria. Yes, this is a malaria*

*I: Yes, three of you had malaria. So, I would like the people who had malaria experience to mention a little bit more about the symptoms of malaria besides having headache.*

*FGD5: It’s as same as this brother said, it’s a fever with a sensation of chill in the body, headache, dizziness [and] nausea. [I] could not do any work [except] sleeping only. Sometimes, vomiting [and] could eat nothing as it troubled [us].*

FGD, male forest-goers aged 27–55

Personal experiences of symptomatic malaria were largely restricted to the past and recent instances of clinical malaria were rare. Older respondents made particular references to the Khmer Rouge period and population movements in the area following the Vietnamese invasion in 1979.

*SSI1: …When I arrived the border, at first, I had malaria.*

*I: For how many years?*

*SSI1: […] that should be [* 19 *] 81 when I had malaria the whole year.*

*I: Oh, about 1981?*

*SSI1: Yes, at that time I was still staff, I was a nurse but we went from mountain to mountain before arriving at the border. [I] didn’t know what types of mosquitos bit me because [I] walked without any clear directions to arrive at the Thai border. So it took a year then I got malaria for also a year… Up to now [I] haven’t have any sickness, [except] liver pain, stomach ache [but] for the liver pain I went to see a Thai doctor [and] have already recovered.*

SSI with 58-year-old female plantation worker

Respondents were keenly aware that malaria was transmitted by mosquito bites, with some reports of ideas from the past about other aetiological factors, which had been recently supplanted by the biomedical explanations given in health education, including that delivered as part of the community engagement for TME. There were references to female mosquitoes and some respondents mentioned the terms “vivax” and “falciparum”, though with little awareness of the similarities and differences between the different species (and this was not a focus of the health education that the TME study provided). They emphasized the use of bed nets as paramount in protecting oneself from mosquito bites and malaria infection. Covering up with clothes and burning fires to create smoke in the forest were also mentioned as ways of repelling mosquitoes.

*I: …Do you know malaria disease?*

*R: Yes, I know falciparum*

*I: Yes, and in this area do you know of the people in the community used the other word instead of malaria?*

*R: I don’t know some said vivax.*

SSI with 53-year-old female plantation worker

Forest-goers were generally identified as at greatest risk of malaria. Men were therefore viewed as at particular risk because they were more likely to spend time in the forest, logging, hunting for bushmeat. This elevated risk of infection was attributed to their greater exposure to mosquitoes and a reduction in bed net usage when in the forests. During FGDs, counter examples were however offered: for example, one regular forest-goer had never experienced malaria, but his wife, who had never visited the forest, had been infected. Newcomers to the area were also seen as at risk; just as, in the past, when they arrived in the area, the now long-term residents had suffered a heavy burden of malaria.

*FGDs: Here mostly men [get malaria].*

*FGD4: [Because] men like going to forest*

*…[…]*

*FGD3: [Men] sleep without hanging bed net. However, [malaria] seem not [happen] to women.*

*FGD4: My husband is also a forest goer but [he] has never had malaria. [However, malaria] mostly happened to me who stay at home (laughing)*

FGD with female community members aged 28–62 years

Respondents emphasized that their treatment seeking focused on the local VMW and the importance of malaria testing. Those who had experienced malaria in the past contrasted the current situation with what they used to do: obtaining drugs from multiple sources, including Thailand and China. Respondents were also familiar with taking multiple-dose anti-malarial regimens; their descriptions of past malaria bouts were also accompanied by references to long periods of treatment and convalesce.

In spite of the scarcity of reports of recent clinical malaria, respondents still expressed concern about malaria and fear of the consequences of infection. This underpinned their malaria prevention efforts.

*I: Yes, and every night time did you use the mosquito net?*

*IDI: Yes, I use the mosquito net.*

*I: Every night?*

*IDI: Yes, I never sleep without mosquito net, I am scared of malaria (laughing)*

IDI with a TME drop-out

For this particular respondent, the fear of malaria infection did not however ensure participation in the three rounds of MDA; becoming sick after the taking the anti-malarial in the first round rather superseded fear of the disease.

### Community engagement

The interviewed TME staff described community engagement in terms of providing education and persuading the local community to participate in the project. They reported a range of activities, such as large village-wide meetings and performances, smaller group meetings and one-to-one meetings with village leaders. Community engagement also entailed collaborating with the local health authority and community leaders, who were involved in the project at an early stage. Indeed, during the first stage of designing the community engagement strategy (after the initial prevalence surveys had taken place), meetings were held with village leaders. The notes that field staff took during these interactions indicate that the leaders’ ideas about what activities would appeal to the community members and encourage them to participate in the MDA were integrated into the project’s community engagement strategy. As part of community engagement, several volunteers were recruited from each village to act as representatives of the project and encourage their neighbours to attend the prevalence survey and MDA.

In spite of the intensive engagement activities that were conducted in each village, study participants’ recollections of activities—particularly the meetings that took place prior to the MDA—were vague, with many haven forgotten details of the events, though, as will be elaborated below, all were aware of the aim of the project.

The study staff recounted challenges that they faced when engaging with the community and attempting to persuade people to participate in the project. These included dealing with the communities’ past experiences of NGOs working in the local area and villagers’ perceptions that they had not kept their promises. This was particularly an issue when, during community engagement activities, there was a misunderstanding about the monetary incentive that study participants would receive. Seemingly, it was not the monetary difference that annoyed community members but rather the sensation that the project was saying one thing and doing another. Another challenge was dealing with concerns about the potential for—through poor needle practices—the spread of HIV/AIDS amongst participants who participated in the prevalence survey. Such fears were based on the locally well-known case that came to light in 2014 of HIV/AIDS being spread through a health provider’s poor needle practices.

*Most of the challenges in village were because of [villagers’] experiences with other NGOs who didn’t keep their promises. And the other thing, for the blood collection, they worry about it spreading other diseases, as in Roka village in Battambang Province, where people got HIV/AIDS through injections. So for that reason, all villagers are scared of blood collection.*

IDI with a female 28-year-old, TME staff member

The community engagement strategy was reactive in its broad approach. The fall in coverage in the second round of MDA (elaborated below) led project staff to follow a different strategy prior to the third round. Staff were concerned that their position as outsiders in the local communities had had a negative impact on coverage. Chiefly, that the meetings held after the first round of treatment did not seem to adequately address villagers’ concerns about side effects. Study staff judged that local leaders would be better able to understand the concerns and be more trusted and credible when answering questions. Therefore, the community engagement staff took a step back from village meetings and representatives from the local health authorities took the lead.

During the semi-structured interviews, respondents did not recall the details of the community engagement activities (and they explained not knowing the details in terms of their forgetfulness), they were well aware of the aim of the project, to “eliminate malaria” (*Lup bam bat chum ngeu krun chaah* ). They were also aware of the main TME activities: blood sampling and giving medicines. Opinions of the project, with regard to its aim of eliminating malaria were generally positive; as indicated, malaria was viewed as a problem in the community (even though there were relatively few recent clinical cases) and participants were pleased that someone was taking action to deal with this issue that had implications in terms of their wellbeing and their economic/subsistence activities.

*Yes it’s very important that they eliminate malaria and we do not get anymore malaria. So we are very happy with this project*

SSI with a 35-year-old female farmer

### Mass anti-malarial administration

As illustrated in Table 1, of the 30 SSI respondents, 12 refused the anti-malarial in round two. However, of these respondents at least six participated and took the anti-malarial in round three (one respondent was excluded from participating on health grounds in round three and one could not be located for interview). The main reasons for refusing to participate in round two or round three were the “side effects” that respondents attributed to the anti-malarial doses. These included vomiting, nausea, shivering, head/stomach ache, dizziness, fatigue/malaise/tiredness, fever, breathing difficulties, stomach cramps and frequent urination.

*Many people got a cold and I got a cold and a headache, and breathing difficulties. In round two, I told the staff that I don’t want to take the medicine and I told [the village leader] as well but I still participated to give blood…*

IDI with a 53-year-old male TME drop-out

Because of the seasonal nature of illness in the site and the fact that TME coincided with the rainy season when a greater proportion of community members suffer minor health complaints, such as common colds and influenza, it is particularly difficult to disentangle the actual from perceived side effects. One respondent even admitted that someone who did not receive the anti-malarial also fell sick. Project staff were well aware of seasonal disease patterns and sought to explain the “side effects” as related to the time of year. Nonetheless, the perceptions of the health impacts of the anti-malarial that they had received in round one seemingly had an impact on participation, particularly in the subsequent round.

*…that one, he did not take the drug yet, but he got sick as well*

SSI with a 52-year-old female farmer

There were suggestions that the experience of side effects depended on one’s underlying state of health. Although, as part of the study, exclusion criteria were applied for existing health complaints (in addition to breastfeeding), some respondents made their own decisions, based on their experience of the anti-malarial, that they were not sufficiently healthy to receive more doses. Also, two respondents mentioned the time of day as relevant to the side effects that they experienced, with evening being the preferred time so that they were able to sleep off the effects.

The reported side effects, in addition to being inconvenient and producing fear amongst respondents simply as threats to health and wellbeing also brought financial concerns. Respondents were worried about having to pay for intravenous (IV) drips—a popular and potentially expensive way of treating minor health complaints, depending on the drip-package that was purchased—to treat their “side effects”. Such drips were purchased from private health providers and then often administered by neighbours or relatives, particularly those who had received healthcare training from the Khmer Rouge. This was in spite of the free healthcare that the project was offering in the study villages, which was explained to villagers during community engagement activities. Villagers described how they chose to purchase IV drips from private health providers in terms of their perceived “energizing” bodily effect. As part of the TME study, which was conducted under the supervision of a medical doctor, such IVs were not offered for complaints of cold or flu-like symptoms because their lack of clinical justification. In addition to the potential monetary costs, respondents were also concerned about the opportunity costs of side effects: that they would be too ill to work on their plantation or in the forests and would forego income.

*My child took this medicine and she got sick. Because of that I spent about 20,0000KHR [*~*5US$].*

IDI with a 60-year-old female TME drop-out

*My child could not take the malaria medicine. He will be troubled if he takes the medicine. From the first round he had three sets of IV fluid injection to get better. For the second round [he] had four sets of IV fluid injection to get bettter. He always gets hot when taking the malaria drug.*

FGD with 27-55-year-old male forest-goers

Nonetheless, many respondents participated in spite of side effects and, indeed, even those who had previously declined the anti-malarial in round two, returned in round three, and took the three-day regimen. Of the 12 SSI respondents who did not participate in round two, six participated in round three. Of these, one had recovered from illness and two had returned to the village after an absence that coincided with round two. For three of the six, their motives for participating in round three despite experiencing side effects after round one and refusing round two were not recorded. This increase in participation was also reflected in the wider coverage and staff speculated that it was a result of changes that were made to community engagement after round two, with local leaders taking a more prominent role in meetings.

Absence also played a role in non-adherence and reduced coverage with community members leaving to work in the forests, on the plantation, further afield in Thailand, or leaving the villages temporarily for other reasons. There were scattered reports of villagers wishing to take the anti-malarials with them to ingest in the forests and a general desire to be warned well in advance of project activities so to schedule their activities in the plantation. Villagers were generally familiar with taking a multi-dose anti-malarial regimen and amongst the 30 SSIs respondents, only two reported missing one of the three daily doses.

Observations in the villages suggested that vocal opponents of TME—some of whom had experienced side effects after round one of the MDA—had influenced family members and neighbours who had also subsequently not participated in MDA round two. The follow-up SSIs also suggested that non-adherence clustered in some families. However, by contrast, in other families, even though one member did not participate in rounds two or there, relatives who did not experience side effects took the anti-malarial.

## Discussion

Several factors influenced coverage and adherence during the mass anti-malarial administration. These focused on the significance of malaria as a local health concern, villagers’ health-seeking behaviour, the health complaints that participants attributed to the administered anti-malarial as “side effects” and the approach of community engagement, particularly in terms of local authority figures leading meetings.

### Malaria as a local health concern

Respondents’ limited reports of recent clinical malaria cases are reflected in local *P. falciparum* incidence data [18]. However, *P. vivax* malaria remains more common in the area [14] and, although some respondents referred to these different species, there was a lack of awareness about what the different infections entailed. The lack of differentiation between the two main species is a potential problem for the TME strategy: *P. vivax* malaria will persist after mass treatment with an ACT (because dormant liver stage parasites will not be killed), and incidences of clinical *P. vivax* in the villages may undermine confidence in mass treatment.

The limited recent reports of malaria contrasted with respondents’ past experiences, particularly when villagers migrated to the area some 30 years ago. Periods of a common, long-lasting and severe illness—and their impact in terms of opportunity costs—seemingly made an impression on the population and ultimately contributed (possibly along with the more recent intensification in malaria control efforts to control artemisinin-resistant parasites [17]) to their continued concern about malaria, which was also reflected in their reported use of bed nets. As was encountered in a recent questionnaire study of factors affecting MDA uptake in Vietnam [23], worries about symptomatic malaria underpinned participants’ willingness to take the anti-malarial, particularly in the first round of MDA. As some cases indicate, these worries were not necessarily sufficient to overcome the impact of health complaints that respondents attributed to the anti-malarial as “side effects” i.e. respondents were worried about malaria but apparently more worried about the “side effects” from the MDA.

### Seasonality, “side effects” and the timing of mass anti-malarial administration

Mass anti-malarial administration was conducted from July to September 2015, during a rainy season. The timing was determined chiefly by earlier delays and the need to begin before the season of peak malaria transmission ended. Many people reported fevers and common colds around the time the study began, and health staff described how colds and fevers are most common during the rainy season. Some respondents’ enthusiasm to participate in subsequent MDA rounds was affected by the health complaints that they attributed to the anti-malarial provided in round one. Concerns about potential “side effects” were also explained in terms of the impact on participants’ ability to work and earn money or them needing to pay for treatments to mitigate the “side effects”. This was also compounded by the fact that the burden of agricultural work is also particularly high during the rainy season.

In the absence of a treatment control group, it is unclear to what extent these complaints resulted from the anti-malarial, or were a largely attributable to the peak in mild illnesses that coincides with the rainy season. Nonetheless, conducting the first rounds of MDA before the rainy season might mitigate the risk of other common illnesses being ascribed to the treatment. It would also reduce the perceived opportunity costs of “side effects”.

The timing of MDA has also been discussed in sub-Saharan Africa: qualitative research in the Gambia highlighted disagreement amongst stakeholders regarding the most appropriate moment in the farming season to administer the anti-malarial [10]. The issue of timing of MDA is particularly relevant when targeting communities with mobile populations, whose movements are often influenced by seasonal harvests. The border regions of countries in the GMS, which are a focus for *P. falciparum* elimination efforts, are also often home to mobile populations and this presents potential challenges for elimination [13, 24]. Table 1 indicates that four respondents were indeed absent for one of the MDA rounds. An in-depth understanding of the patterns of migration and movement are therefore necessary for the planning of MDA.

### “Side effects” and health seeking behaviour

The impact of the “side effects” were also compounded by the general demand for pharmaceuticals, particularly IVs for relatively minor complaints, which villagers purchased at significant cost from local private vendors. To deal with the “side effects”, there were scattered reports of villagers purchasing IV “kits” (comprising saline solution, and sometimes antibiotics and “vitamins”), in spite of the available free healthcare provided by the project. The fears were more related to the possibility of having to pay for an IV in case of “side effects”, which, in light their experiences after round one, some participants viewed as likely. And, with many older villagers having received basic medical training as part of their past involvement with the Khmer Rouge, someone was often on hand to administer the IVs without the assistance of health providers.

Elsewhere in Cambodia and various Asian contexts, IVs have been highlighted as a popular remedy for complaints, including malaria [13, 24, 25]. The popularity of this method of delivering usually saline and sometimes antibiotic (and injections in general) across Asia has been explained in terms of its similarities with acupuncture and also the widespread use of injections by barefoot doctors in rural China during the middle of the twentieth century [26]. In this study, respondents explained their preference for IV in terms of its energizing bodily effect. Anthropologists have also highlighted the importance of how perceived side effects from anti-malarials are interpreted in terms of local understanding of illness and healing [27]. Given their popularity across the region, community engagement linked to mass anti-malarial administration must therefore take into account the potential demand for IVs in response to perceived side effects.

Villagers’ readiness to use a range of medicines with little advice from healthcare providers contrasted with their reports of universally consulting VMWs, in the case of suspected malaria infection. This juxtaposition suggests that further detailed research—encompassing long-term observation methods—is needed to gain a reliable understanding of anti-malarial use in the area. This is particularly important given the role that the surrounding area has played in the emergence of artemisinin resistance [17] and the sole reliance on treatment records from the VMW system to establish incidence in an area where *P. falciparum* elimination is planned.

### Understandings of mass anti-malarial administration

The interviews suggested that villagers were aware of the projects’ main activities and the overall aim, with the Khmer term for malaria elimination extremely well known. However, they were less familiar with the details of the study. This is unsurprising given the complex rationale for eliminating a disease in an area with a relatively low current clinical incidence to prevent catastrophe half-way around the world. Even educated members of the project team struggled to get to grips with the scientific basis for the project. It is notable that, in spite of this, villagers still participated, particularly in rounds one and three. This suggests that although providing detailed information is important, particularly in terms of meeting the ethical requirements of informed consent, the simple message of “malaria elimination” was what resonated amongst villagers.

### Flexibility of the community engagement strategy

Study staff were acutely aware of the decline in coverage that occurred between rounds one and two—from around 90 to 72% [28]. They recognized that the “side effects” played a role in this and that their explanations regarding the source of these health complaints—as normal seasonal illnesses—had not resonated with all villagers. Therefore, they sought to take a different approach to community engagement prior to round three. This entailed local leaders and health staff taking more prominent roles in community meetings; although present, staff from the implementing organization did not take the lead in explaining the intervention. After this change in strategy the round three coverage increased to above 85% [20]. The importance of community leaders taking actives and visible roles in explaining MDA has also been reported in the Gambia [10] and has been recognized as a principal of community engagement for infectious disease research in general [29]. Indeed, the community taking a leading role in directing how MDAs—and other interventions—are implemented has been acknowledged as underpinning sustainable malaria elimination programmes [30].

## Strengths and limitations

Although limited by the clinical trial context in which the data were collected, the findings offer some useful signposts for the design of future programmes of mass anti-malarial administration in the region. Moreover, the study design used multiple methods, multiple respondents and a team of two data collectors to allow triangulation and to reduce the potential bias that a single data collection technique or respondent might produce. Randomly selecting SSI participants meant that attitudes from across the target communities were included and indeed a point of theoretical saturation (whereby no further novel findings were encountered) was reached within the 30 interviews. The use of village leaders to select participants for focus group participants was unavoidable because of the need to identify and contact hard-to-reach groups, particularly those who work in the local forests. The addition of observations of community engagement meetings and other study-related events in the communities meant that reported behaviours could be interrogated. This was particularly important because the staff who conducted interviews and questionnaires would be associated with the clinical study and this may have influenced some respondents to respond favourably with regard to their reported attitudes towards TME. Villagers were however willing to complain about the side effects of the anti-malarial, some aspects of the project, such as the incentives, and ready to discuss nonadherence.

## Conclusion

Incidence of clinical malaria is low in the study area but it remains a health concern because of past experiences and the opportunity costs that a bout of malaria entails. This, combined with a community engagement strategy, which presented a simple message about an intervention that could address these concerns, contributed to high coverage in round one of anti-malarial administration. The peak in seasonal health complaints coincided with the administration of the anti-malarial and amplified complaints of “side effects”. This contributed to a reduced participation in round two, in spite of the explanations that study staff offered. After the change in focus of engagement activities prior to round three, coverage levels increased. Careful planning is therefore needed with regard to the timing of mass anti-malarial administrations in the context of seasonal illness (and mobility) patterns. An approach to community engagement, which involved community members taking prominent roles and prioritizing a single simple message seemingly had a positive impact on coverage. Further research is needed to investigate the factors that impact coverage in scenarios that more closely resemble implementation i.e. without participation incentives, blood sampling and free healthcare.
